# Racial Differences in Spinal Cord Compression Related Hospitalizations in Patients with Multiple Myeloma

**DOI:** 10.1007/s44228-023-00027-9

**Published:** 2023-02-03

**Authors:** Samer Al Hadidi, Deepa Dongarwar, Carolina Schinke, Sharmilan Thanendrarajan, Maurizio Zangari, John D. Shaughnessy, Frits van Rhee

**Affiliations:** 1 Myeloma Center, Winthrop P. Rockefeller Cancer Institute, University of Arkansas for Medical Sciences, Little Rock, AR USA; 2 McGovern School of Medicine, The University of Texas at Houston, Houston, TX USA

**Keywords:** Multiple myeloma, Disparity, African American, Black persons, Spinal cord compression, Hispanics

Non-Hispanic (NH) Black and Hispanic persons with multiple myeloma (MM) face multiple health-related disparities that include lower access to novel therapies and under-utilization of autologous stem cell transplantation [1, 2]. Differences in biology may be associated with different clinical presentations and/or outcomes [3, 4]. MM is more common in NH Black persons. Spinal cord compression (SCC) is a medical emergency for which MM was reported to be the third most prevalent underlying cancer diagnosis associated with SCC hospitalizations [5]. Racial/ethnic differences in the rates of SCC in patients with MM were not previously reported. Our study aimed to study if such differences exist for patients with MM admitted with SCC in the U.S.

We conducted a 12-year retrospective analysis of inpatient hospitalizations from 2008 to 2019 using the Nationwide Inpatient Sample (NIS) data, provided by the Healthcare Cost and Utilization Project (HCUP), sponsored by the Agency for Healthcare Research and Quality. The study sample included the occurrence of MM in the discharge records of adults (18 + years). The primary end-point of the study was SCC. The ninth and tenth editions of the international classification of diseases (ICD-09 and ICD-10, respectively) were used to identify MM and SCC diagnoses in patient records. MM was identified using ICD-09 codes—203.xx and ICD-10 codes—C90.xx, whereas SCC was identified using ICD-09 codes—336.9, 733.90, V12.49 and ICD-10 codes—G95.20, C79.51, Z86.69.

The covariates included in this study were patient socio-demographic characteristics such as patients’ age—categorized into 18–34, 35–49, 50–64, 65 + years; race/ethnicity – categorized as NH White, NH-Black, Hispanic, other; gender—female or male; zip code–based median household income—lowest, 2nd, 3rd and highest quartiles; primary insurance—Medicare, Medicaid, private insurance, self-pay, other; hospital characteristics such as bed size—classified as small, medium and large; hospital location and teaching status—grouped as rural, urban teaching and urban non-teaching; hospital region—Northeast, Midwest, South and West.

The analysis for this study was generated using R version 3.5.1 (University of Auckland, Auckland, New Zealand), R Studio Version 1.1.423 (Boston, MA). Due to the de-identified nature of the NIS, the University of Arkansas for Medical Sciences (UAMS) Institutional Review Board (IRB) classified this study as being exempted from IRB approval. We conducted descriptive statistics to examine the patient and hospital characteristics associated with MM and SCC in MM hospitalizations. We also calculated the prevalence of MM-related hospitalizations and SCC in MM cases. All of the hospitalizations were weighted to account for the complex sampling design of the NIS, which allowed for the generation of national estimates.

To identify and describe temporal changes in the rates of SCC in MM-related hospitalizations during the 12-year study period, joinpoint regression was utilized. Each joinpoint indicated a change in the trend, and an average annual percent change (AAPC) and 95% confidence interval (CI) were estimated to characterize how the rate of the outcome (SCC in MM cases) changed during the entire study duration. Lastly, adjusted survey logistic regression models were utilized to examine the hospitalization factors associated with SCC. All patient and hospital characteristics were used for adjustment in the model. All statistical tests were two-tailed with type-I error rate set at 5%.

Of 368,188,112 hospitalizations, 1,247,364 (0.34%) were related to MM. These were more prevalent in older adults, males, and NH-Blacks. Of MM hospitalizations, 56,902 (4.6%) were associated with SCC diagnosis.

MM hospitalizations were more common in NH Blacks (5 per 1000 hospitalizations) compared to NH-Whites (3.3 per 1000 hospitalizations) and Hispanics (2.6 per 1000 hospitalizations). SCC-related hospitalizations in patients admitted with MM were more common in NH-Whites (4.9 per 100 MM hospitalizations) compared to NH-Blacks and Hispanics (4.2 and 4 per 100 MM hospitalizations, respectively) (Table 1).

**Table 1:** Characteristics of multiple myeloma and spinal cord compression hospitalizations

	Overall	Multiple Myeloma		Spinal cord compression	
	*N* = 368,188,112	*N* = 1,247,364, % = 0.34	Prevalence per 1000 hospitalizations	*N* = 56,902, % = 4.6	Prevalence per 100 multiple myeloma hospitalizations
Age					
18–34 years	71,186,975	4391	0.1	154	3.5
35–49 years	55,955,733	66,129	1.2	1882	2.8
50–64 years	92,401,226	386,176	4.2	13,910	3.6
65 + years	148,644,178	790,668	5.3	40,955	5.2
Race/ethnicity					
NH-White	232,165,261	756,239	3.3	36,816	4.9
NH-Black	50,463,485	254,780	5.0	10,686	4.2
Hispanic	36,554,986	93,738	2.6	3753	4.0
NH-Other	21,215,167	60,445	2.8	2653	4.4
Missing	27,789,212	82,164	3.0	2995	3.6
Gender					
Male	151,625,231	681,138	4.5	30,702	4.5
Female	216,371,220	565,843	2.6	26,170	4.6
Missing	191,661	385	2.0	30	7.8
Zip Income quartile					
Lowest quartile	107,364,203	328,946	3.1	14,424	4.4
2nd quartile	94,086,154	299,811	3.2	13,112	4.4
3rd quartile	85,746,861	300,416	3.5	14,004	4.7
Highest quartile	72,973,160	294,965	4.0	14,507	4.9
Missing	8,017,734	23,228	2.9	855	3.7
Primary payer					
Medicare	170,390,168	832,941	4.9	38,281	4.6
Medicaid	61,543,837	76,271	1.2	2858	3.7
Private Insurance	104,863,205	291,740	2.8	12,026	4.1
Self-Pay	30,706,819	44,144	1.4	3604	8.2
Missing	684,083	2270	3.3	133	5.9
Disposition					
Routine	240,913,151	645,493	2.7	6359	1.0
Transferred	66,300,428	280,060	4.2	16,120	5.8
Died	8,099,712	65,646	8.1	20,278	30.9
DAMA	4,837,844	5898	1.2	80	1.4
Other	47,809,172	249,413	5.2	14,010	5.6
Missing	227,805	856	3.8	55	6.4
Hospital bed size					
Small	59,596,686	174,693	2.9	7680	4.4
Medium	99,144,773	303,922	3.1	14,167	4.7
Large	208,082,480	765,079	3.7	34,920	4.6
Missing	1,364,173	3671	2.7	135	3.7
Hospital location and teaching status					
Rural	39,229,092	94,723	2.4	3680	3.9
Urban non-teaching	119,375,005	323,358	2.7	13,621	4.2
Urban teaching	208,219,843	825,614	4.0	39,466	4.8
Missing	1,364,172	3671	2.7	135	3.7
Hospital region					
Northeast	70,796,846	264,720	3.7	11,693	4.4
Midwest	83,671,391	287,726	3.4	12,672	4.4
South	143,348,402	476,550	3.3	21,636	4.5
West	70,371,473	218,369	3.1	10,901	5.0

AAPC showed an uptrend for all racial/ethnic groups and was higher in NH-Blacks when compared to both Hispanics and NH-Whites (10.5, 9.6 and 7.9, respectively *p* < 0.01) (Fig. 1). Despite the higher AAPC in NH- Blacks and Hispanics, NH-Whites continued to have the highest rate of SCC in MM hospitalizations. Multi-variable adjusted survey logistic regression for patient’s gender, age, income quartile, primary payer, and comorbidities showed lower odds ratio (OR) of SCC in NH-Blacks (OR 0.83 (95% CI 0.78–0.88) and Hispanics (OR 0.83 (95% CI 0.76–0.91) when compared to NH-Whites.

**Fig. 1:**
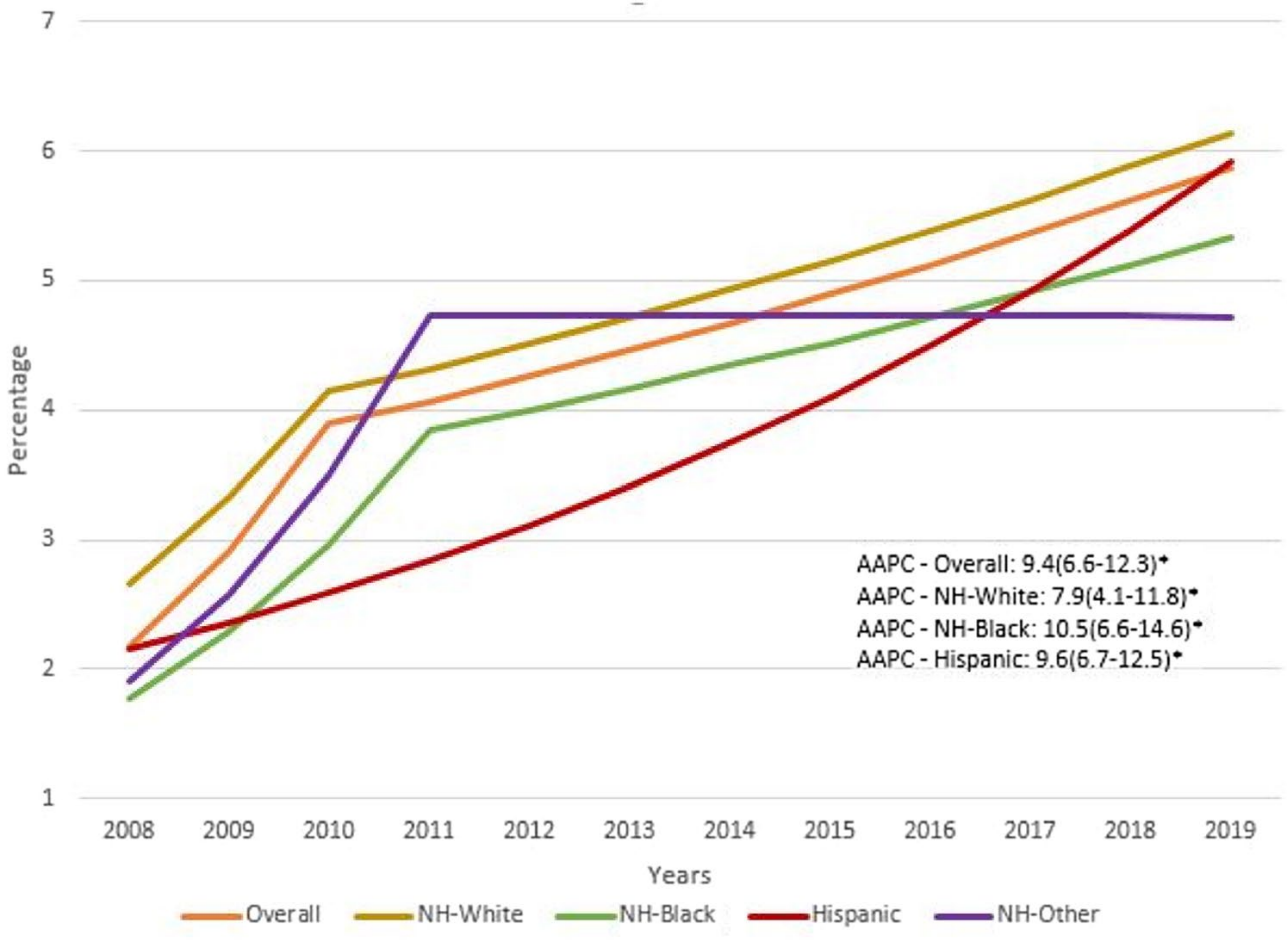
Trends in the rates of spinal cord compression in multiple myeloma hospitalizations 2008–2019

Our study is the first to report on racial/ethnic differences of the rates of SCC in hospitalized MM patients using a representative large national sample. MM is a heterogeneous disease, and such clinical difference can be related to variability in the molecular subtype of MM and/or other clinical features of MM. Future analysis from the UAMS MM database is being performed to help in exploring biological differences in different racial/ethnic groups according to the molecular MM subtype and/or clinical presentation.

We found that the rates of SCC in patients with MM are overall increasing and is lower in NH-Blacks and Hispanics when compared to NH-Whites. SCC is a medical emergency that can result in pain and potentially irreversible loss of neurologic function. Extradural SCC can be related to extramedullary plasmacytoma or a bone fragment secondary to a pathological vertebral fracture. Early detection and intervention may help in avoiding permanent paraplegia. The increased rates of SCC in patients with MM found in our study may be related to better recognition and/or diagnosis of SCC. Other potential explanations may be related to the change in treatment landscape and/or longer survival of MM patients.

Using data from the NIS has some limitations, such as lack of individual patient data, inability to categorize other minor ethnic groups or patients who self-identify as multi-racial and the consideration of recurrent hospitalizations as distinct observations. Moreover, data on outpatient consultations are not available and the recognition of the diagnosis of SCC in the outpatient setting is increasing [6].

Our analysis showed that the rates of SCC in patients with MM are overall increasing and is lower in NH-Blacks and Hispanics when compared to NH-Whites. This may be related to different biology in different ethnic/racial groups which needs to be further explored in future studies.

## Data Availability

The data that support the findings of this study are available upon request to the corresponding author. The data are not publicly available due to privacy or ethical restrictions.
